# Comparative analysis of hyperuricemia induction methods and probiotic interventions in mice

**DOI:** 10.1016/j.crmicr.2025.100422

**Published:** 2025-06-12

**Authors:** Yanbo Wang, Huijiao Zhang, Shujun Liu, Sheng Sun, Weibin Ren, Tao Wang, Shujuan Zhang, Hangping Yao, Changzhong Jin, Nanping Wu

**Affiliations:** a Jinan Microecological Biomedicine Shandong Laboratory, Jinan 250117, PR China; b Changchun Veterinary Research Institute, Chinese Academy of Agricultural Sciences, Changchun 130122, PR China; c State Key Laboratory for Diagnosis and Treatment of Infectious Diseases, The First Affiliated Hospital, College of Medicine, Zhejiang University, No 79 Qingchun Road, Hangzhou 310003, Zhejiang Province, PR China

**Keywords:** Probiotics, Hyperuricemia, Mouse model, Uric acid metabolism, Renal function

## Abstract

Probiotics are widely used as functional food additives, but more studies are needed for their use in mitigation of hyperuricemia (HUA). Currently, there are no standardized animal models for HUA. There is significant variability in the animal strains, drugs, dosages, and modeling periods used in published studies, which greatly impacts the comparability of experimental results and complicates the effectiveness evaluation of therapeutic agents. In this study, we systematically compared different methods for inducing HUA in C57BL/6JNifdc mice and investigated the potential of probiotics to regulate blood uric acid levels. Our results revealed significant differences in blood uric acid levels and renal impairment among the methods. Adenine causes severe renal injury, increases hepatic xanthine oxidase and purine nucleoside phosphorylase activity, and inhibits the expression of uric acid transporter proteins in the kidneys. In contrast, inosine, guanosine, and hypoxanthine mildly induce hyperuricemia in mice. We screened seven probiotic strains from fermented dairy products and evaluated their effects on uric acid metabolism both in vitro and in vivo. Notably, probiotics showed significant potential for degrading uric acid, modulating the expression of uric acid transporter proteins in the kidneys, and inhibiting renal inflammation. These findings provide valuable insights into the pathogenesis of HUA and highlight the therapeutic potential of specific probiotics for managing this metabolic disorder. Our study highlights the importance of selecting appropriate animal models for HUA research and suggests that probiotics can be used as promising functional food additives to assist in hyperuricemia treatment.

## Highlights

• Different HUA modeling methods show differences in disease phenotypes and liver/kidney functions.
• Adenine induces severe renal injury, while inosine/guanosine models mimic mild HUA.
• Probiotics suppress renal URAT1/GLUT9 expression and inflammation.
• Probiotics modulate gut SCFAs, correlating with renal protection and UA synthesis.

## Graphical abstract

**Figure:**
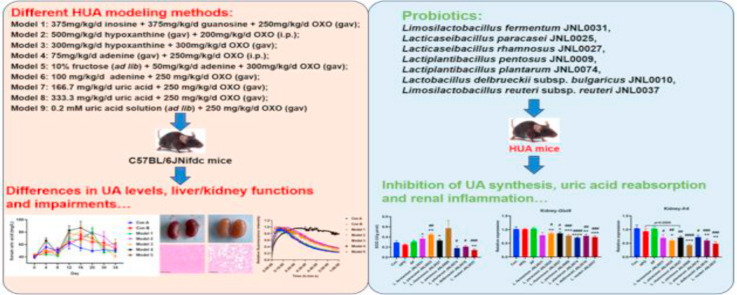

## 1 Introduction

Hyperuricemia (HUA), a metabolic disorder resulting from impaired purine metabolism and disrupted uric acid excretion, is increasing globally (Dalbeth et al., 2021; Ichida et al., 2012). It has now become the second most prevalent metabolic disease after type II diabetes and is increasingly affecting younger populations. According to statistical data from 2020, there are approximately 980 million HUA patients worldwide, and the patient number is projected to 1.42 billion by 2030. HUA not only directly causes gout but also lead to a range of serious health issues, including renal and cardiovascular diseases, and metabolic syndrome (Wan et al., 2016; Wu et al., 2017). Therefore, investigating the pathogenesis of HUA and developing effective prevention and treatment strategies are crucial.

An imbalance between uric acid synthesis and excretion is the underlying cause of HUA. Uric acid synthesized by the body is excreted by two main pathways: renal and intestinal. Approximately two-thirds of uric acid is excreted from the body in the urine through the kidneys, and the rest is excreted into the intestines and metabolized. Gut microbes play a significant role in maintaining purine homeostasis and regulating serum uric acid levels. Many intestinal commensal bacteria can metabolize uric acid under anaerobic conditions, converting it to xanthine, lactic acid, or short-chain fatty acids (SCFAs) (Liu et al., 2023). Additionally, the gut microbiota can use purines as carbon and energy sources, thereby influencing the overall homeostasis of uric acid and other purines (Kasahara et al., 2023). Gut microbes can also regulate uric acid excretion by affecting the expression of uric acid transporter proteins, such as ATP-binding cassette subfamily G member 2 (ABCG2) and solute carrier family 2 member 9 (SLC2A9, also known as GLUT9), in the intestine and kidneys (Xu et al., 2024; Zhang et al., 2022). The gut microbiota composition in HUA and gout patients differs from that in healthy individuals and is characterized by reduced abundance and diversity, which may significantly alter amino acid and nucleotide metabolic pathways (Wei et al., 2022). These findings highlight the important role of the gut microbiota in the development of HUA, suggesting that modulating the gut microbiota could be a novel therapeutic strategy for HUA.

Currently, the clinical treatment regimens for HUA primarily involve drug therapy combined with strict dietary management to regulate blood uric acid levels. However, >20%-30 % of gout patients struggle to achieve the desired uric acid control with existing medications (such as febuxostat, benzbromarone and allopurinol). Moreover, these medications have side effects and are not suitable for long-term daily use. On the other hand, a strict low-purine diet can significantly impact patients' quality of life and lead to poor compliance. Many studies have shown that probiotics, such as *Lactiplantibacillus plantarum, Akkermansia muciniphila* and *Lacticaseibacillus rhamnosus*, play key roles in regulating uric acid synthesis, catabolism, and excretion (M. Li et al., 2023; Zhang et al., 2022; H. Zhao et al., 2022). Probiotics are safe and can enhance intestinal functional homeostasis, offering a new option for HUA treatment.

The use of animal models is essential for studying the pathogenesis treatment options for HUA. However, there is currently no standardized model for HUA. HUA animal models can be divided into two main categories: genetically induced models and environmentally induced models (Lu et al., 2019). Genetically induced HUA mouse models are not suitable for large-scale drug screening because of their complex technology, high cost and high mortality (Lu et al., 2019). Environmentally induced HUA models are the most commonly used and focus on two core strategies: (1) increasing uric acid synthesis through supplementation with uric acid or its precursors and (2) inhibiting uricase activity, thereby reducing uric acid catabolism. The animal strains and modeling methods used in environmentally induced HUA models vary widely, leading to inconsistent study results and making it difficult to accurately evaluate the effectiveness of therapeutic regimens. Moreover, the reported HUA animal models do not demonstrate long-term changes in blood uric acid, which makes it difficult to obtain additional valuable references.

In this study, we first utilized C57BL/6JNifdc mice to systematically compare the differences in blood uric acid concentration trends and liver and kidney function among mice subjected to several different modeling methods. We then conducted in vitro experiments to screen for probiotics with uric acid-lowering potential. The effects and mechanisms of various probiotics in lowering uric acid were also investigated using the HUA mouse model. This study provides valuable insights into modeling HUA in mice and the clinical application of probiotics for treating HUA.

## 2 Materials and methods

### 2.1 Isolation, identification and culture conditions of probiotics

The probiotics used in this study were isolated from fermented dairy products sourced from Inner Mongolia, Qinghai Province, and Xinjiang, China. Initially, the samples were subjected to gradient dilution with saline and plated onto solid MRS (De Man, Rogosa and Sharpe) media. The plates were then incubated under anaerobic conditions at 37 °C for 48 h. Single colonies were selected and purified through two rounds of cultivation. For species identification, the 16S rRNA gene of the bacteria was amplified using the primers 27F (5′- AGAGTTTGATCCTGGCTCAG-3′) and 1492R (5′- GGCTTACCTTGTTACGACTT-3′). The gene sequences were obtained via Sanger sequencing. A phylogenetic tree was constructed using the neighbor-joining method with MEGA6 software (Tamura et al., 2013).

### 2.2 Analyzing the uric acid degradation ability of probiotics in vitro

Probiotics were cultured in MRS medium at 37 °C for 16 h. The bacteria were then collected by centrifugation at 4 °C and 6000 rpm for 5 min. The pellet was washed twice with HEPES buffer (50 mM, pH 7.0) and resuspended in 1 mL of uric acid solution (1 mM uric acid in HEPES buffer) to contain approximately 10^9^ CFU of bacteria. The suspension was incubated at 37 °C with shaking at 100 rpm for 2 h. The reaction was terminated by heating the samples at 100 °C for 10 min. For the negative control group, the bacterial suspension was first heated at 100 °C for 10 min before being incubated with shaking at 37 °C for 2 h. The supernatant was collected by centrifugation at 6000 rpm for 5 min at room temperature. The uric acid content in the supernatant was measured via a Uric Acid Colorimetric Assay Kit (E-BC-K016-M, Elabscience, China) according to the manufacturer's instructions. The uric acid degradation rate was calculated according to the following formula: uric acid degradation rate = (1 - remaining uric acid/total uric acid) × 100 %.

### 2.3 Analyzing the ability of probiotics to degrade guanosine in vitro

Probiotics were cultured in MRS medium at 37 °C for 16 h. The bacteria were collected by centrifugation at 4 °C and 6000 rpm for 5 min. The pellet was washed twice with PBS (pH 7.4) and resuspended in 2 mL of guanosine solution (0.5 mM in PBS) containing approximately 10^9^ CFU of bacteria. The suspension was incubated at 37 °C with shaking at 100 rpm for 2 h. The bacterial suspension was then filtered through a 0.22 μm pore-size filter membrane, and the supernatant was collected. The *Lactobacillus casei* strain Shirota was used as a control strain. The contents of guanosine and guanine in the supernatant were analyzed using an Agilent 1260 Infinity II. An Agilent ZORBAX SB-Aq column was used, with the mobile phase consisting of an aqueous solution of ammonium formate (10 mM, pH=3.3 adjusted with formic acid) containing 1 % methanol. The sample injection volume was 20 μL, and the flow rate of the mobile phase was 1 mL/min. The residual guanosine and guanine contents were calculated on the basis of the peak area. The guanosine conversion rate and guanosine removal rate were determined using the following formulas: guanosine conversion rate = (1 - residual guanosine/total guanosine) × 100 %; and guanosine removal rate = [1 - (residual guanosine + guanine)/total guanosine] × 100 %.

### 2.4 Mice and treatment

(1) Comparison of different methods for inducing hyperuricemia

Forty-two male C57BL/6JNifdc mice (8 weeks old, weighing 21–24 g) were randomly divided into seven groups (*n* = 6 in each group) after one week of acclimatization. The mice in each group were treated as follows: **Con A group**, 200 μL of 0.5 % carboxymethylcellulose sodium (CMC—Na) solution by gavage; **Con B group**, 200 μL of 0.5 % CMC—Na solution by gavage, along with 100 μL of saline intraperitoneally; **Model 1 group**, 200 μL of a mixture of inosine (375 mg/kg/d), guanosine (375 mg/kg/d) and oteracil potassium (OXO, 250 mg/kg/d) by gavage (D. Li et al., 2023); **Model 2 group**, 200 μL of hypoxanthine (500 mg/kg/d) by gavage and 100 μL of OXO (200 mg/kg/d) injected intraperitoneally (Ni et al., 2021); **Model 3 group**, 200 μL of a mixture of hypoxanthine (300 mg/kg/d) and OXO (300 mg/kg/d) by gavage (Shi et al., 2023); **Model 4 group**, 200 μL of adenine (75 mg/kg/d) by gavage, and 100 μL of OXO (250 mg/kg/d) intraperitoneally (H. Zhao et al., 2022); and **Model 5 group**, 200 μL of a mixture of adenine (50 mg/kg/d) and OXO (300 mg/kg/d) by gavage, with ad libitum access to a 10 % fructose solution (Meng et al., 2023). The mice were weighed at regular intervals, and blood was collected from the retro-orbital venous plexus after a 12-hour fast. After 34 days of treatment, the mice were anesthetized and sacrificed. Mouse tissues were collected for subsequent analysis. Owing to the poor health status of the mice caused by adenine, animals in the Model 4 group were terminated on Day 20 for humanitarian reasons.

Additionally, we tested four alternative modeling methods: (1) 200 μL of a mixture of OXO (250 mg/kg/d) and adenine (100 mg/kg/d) by gavage; (2) 200 μL of a mixture of OXO (250 mg/kg/d) and uric acid (166.7 mg/kg/d) by gavage; (3) 200 μL of a mixture of OXO (250 mg/kg/d) and uric acid (333.3 mg/kg/d) by gavage; and (4) 200 μL of OXO (250 mg/kg/d) by gavage, with ad libitum access to a 0.2 mM uric acid solution. Each group consisted of five male C57BL/6JNifdc mice (8 weeks old, weighing 21–24 g). The mice were weighed at regular intervals, and blood was collected from the retro-orbital venous plexus after a 12-hour fast.

(2) Treatment of hyperuricemia model mice with probiotics

Seventy male C57BL/6JNifdc mice (8 weeks old, weighing 21–24 g) were randomly divided into ten groups (*n* = 7 in each group) after one week of acclimatization. The mice in each group were treated as follows: **Con group**, 200 μL of 0.5 % CMC—Na solution by gavage; **HPD group**, 200 μL of a mixture of hypoxanthine (300 mg/kg/d) and OXO (300 mg/kg/d) by gavage; **AP group**, 200 μL of allopurinol solution (5 mg/kg/d, dissolved in saline) by gavage, based on the HPD group; and **Pro 1 to Pro 7 groups,** gavage with probiotics *Limosilactobacillus fermentum* JNL0031, *Lacticaseibacillus paracasei* JNL0025, *Lacticaseibacillus rhamnosus* JNL0027, *Lactiplantibacillus pentosus* JNL0009, *Lactiplantibacillus plantarum* JNL0074, *Lactobacillus delbrueckii* subsp. *bulgaricus* JNL0010 and *Limosilactobacillus reuteri* subsp. *reuteri* JNL0037, respectively, based on the HPD group. Each probiotic strain was administered at a dose of 2 × 10^9^ CFU per day. The mice were weighed every five days, and blood was collected from the retro-orbital venous plexus after a 12-hour fast. On Day 16, the mice were anesthetized and sacrificed. Kidney, liver, and small intestine tissues were collected for subsequent analysis.

The probiotics used in this study have been deposited in the China General Microbiological Culture Collection Center (CGMCC) under the numbers CGMCC No 33,217, 31,043, 33,216, 31,041, 31,047, 31,042 and 33,218. The animal experiments were approved by the Animal Experimental Ethical Inspection of the Jinan Microecological Biomedicine Shandong Laboratory. All applicable international, national, and institutional guidelines for the care and use of animals were followed.

### 2.5 Biochemical analysis

The uric acid levels in the serum, urine and feces were measured using a Uric Acid Colorimetric Assay Kit (E-BC-K016-M, Elabscience, China). Serum levels of creatinine, urea, alanine aminotransferase (ALT), and aspartate aminotransferase (AST) were determined using the Creatinine Colorimetric Assay Kit (E-BC-K188-M, Elabscience, China), Urea Colorimetric Assay Kit (E-BC-K183-M, Elabscience, China), Alanine Aminotransferase (ALT/GPT) Activity Assay Kit (E-BC-K235-M, Elabscience, China) and Aspartate Aminotransferase (AST/GOT) Activity Assay Kit (E-BC-K236-M, Elabscience, China), respectively. Xanthine oxidase (XOD) and purine nucleoside phosphorylase (PNP) activity in the liver were assessed using the Xanthine Oxidase Activity Assay Kit (E-BC-K805-M, Elabscience, China) and Alkaline Phosphatase Assay Kit (P0321S, Beyotime, China), respectively.

### 2.6 Histological analysis

In this study, liver and kidney tissues were fixed in 4 % paraformaldehyde and processed. Paraffin sections were prepared and stained with hematoxylin‒eosin (H&E) to visualize the tissue structure. The renal index was calculated as the ratio of kidney weight to body weight.

### 2.7 Detection of renal filtration function in mice

The glomerular filtration rate (GFR) of the mice was measured using the MediBeacon Transdermal GFR (Mini, MediBeacon, Germany) (Dorshow et al., 2024). Briefly, the hair on the back of each mouse was removed the day before the experiment without damaging the skin. A solution of the tracer FITC-Sinistrin (35.0 mg/mL) was prepared in saline before the experiment. The mice were then anesthetized by inhalation and fitted with a MediBeacon Transdermal GFR detector. The FITC-Sinistrin solution (7 mg/100 g body weight) was subsequently injected into the tail vein, and the fluorescent signal was monitored for 60–90 min. Finally, the GFR was calculated on the basis of the clearance half-life of FITC-Sinistrin using the following formula: $GFR\;\left[\mu L/min/100gbody\;weight\right]=\frac{14616.8[\mu L/100g\;body\;weight]}{t1/2(FITC-Sinistrin)[min]}$.

### 2.8 Quantitative real-time PCR (RT‒qPCR) analysis

Total RNA was extracted from the kidney and small intestine using RNAisoPlus (TaKaRa Code No 9109, Dalian, China). The One-Step TB Green® PrimeScript™ RT‒PCR Kit II (TaKaRa Code No RR086A, Dalian, China) was used to determine gene transcription levels. The reactions were performed using a Roche LightCycler 480II Real-Time PCR Detection System. The sequences of primers used were Primer-BLAST (https://www.ncbi.nlm.nih.gov/tools/primer-blast/index.cgi?LINK_LOC=BlastHome) or by referring to a previous study (Jin et al., 2018). The primer sequences are listed in Supplementary Table S1. β-Actin was used as a reference gene. The PCR and amplification conditions were set according to the manufacturer's recommended optimal conditions. The relative expression of target genes was calculated using the 2^-∆∆Ct^ formula.

### 2.9 Detection of short-chain fatty acids in colon contents

An appropriate sample of colon contents was collected and precisely weighed. Sample pre-treatment and gas chromatography mass spectrometry (GC–MS) analysis procedures were referenced in the published literature (Han et al., 2024).

### 2.10 Statistical analysis

The data are expressed as the means ± standard errors of the means (SEMs). A *t*-test was used for significant difference analysis. A value of *p*< 0.05 was considered statistically significant. Correlations between indicators were analyzed using Pearson correlation analysis. Figures were plotted using GraphPad Prism version 7.00 for Windows (GraphPad Software, La Jolla California USA; www.graphpad.com).

## 3 Results

### 3.1 Effects of different methods on inducing hyperuricemia in mice

**Fig. 1:**
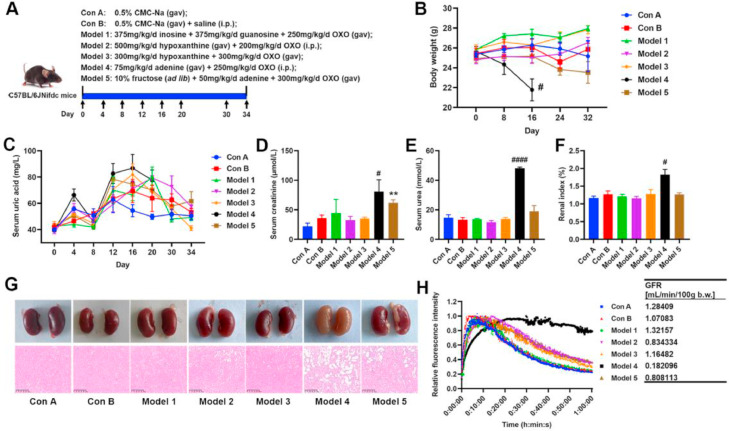
**Comparison of blood uric acid levels and renal tissue damage in different HUA modeling methods.** (A) Schematic diagram of the study design. (B) Body weight. (C) Serum uric acid. (D) Serum creatinine. (E) Serum urea. (F) Renal index. (G) Photographs and H&E staining of the kidneys. (H) Glomerular filtration rate (GFR) of mice. Data represent mean values ± SEM (*n* = 6 per group). Statistical analysis was performed using unpaired T test. The symbol “*” indicates comparison with Con A group. **p < 0.01. The symbol “**#**” indicates comparison with Con B group. **#***p* < 0.05, **####***p* < 0.0001.

In this study, we compared various methods for inducing HUA in C57BL/6JNifdc mice on the basis of literature reports (Fig. 1A). The results revealed that the body weights of the mice in the Model 1, Model 2, Model 3 and Model 5 groups did not significantly differ from those in the control group. However, the body weights of mice in the Model 4 group continued to decrease. On the 16th day of the experiment, the body weights of the mice in the Model 4 group were significantly lower than those in the Con B group (Fig. 1B). Additionally, the mice in the adenine (100 mg/kg/d) model group died within 3 days, suggesting significant toxicity at high concentrations of adenine (data not shown). Overall, the serum uric acid levels in the mice initially increased but then decreased (Fig. 1C). Specifically, mice in the Model 1 group presented significantly higher serum uric acid levels between Days 12 and 20, ranging from 11.5 % to 62.0 % higher than those in the Con A group. By Day 30, the serum uric acid levels in the Model 1 mice were no longer significantly different from those in the control group. The mice in the Model 2 group presented higher serum uric acid levels than those in the Con B group did between Days 20 and 34, with increases ranging from 7.9 % to 24.4 %. The mice in the Model 3 group had higher serum uric acid levels than those in the Con A group did between Days 12 and 30, ranging from 8.6 % to 50.5 % higher. Mice in the Model 4 group had significantly higher serum uric acid levels than those in the Con B group did on Day 4, but these levels decreased by Day 8 and were no longer significantly different from those in the other groups. Between Days 12 and 20, the serum uric acid levels in the Model 4 group were 21.6 % to 30.2 % higher than those in the Con B group. Owing to the toxicity of adenine, the vital signs of the mice in the Model 4 group were poor, leading to termination of the experiment on Day 20 for ethical reasons. The serum uric acid levels of the mice in the Model 5 group were 12 % to 41 % higher than those in the Con A group from Days 12 to 34 (Fig. 1C). Additionally, neither the gavage of uric acid nor the addition of uric acid to the drinking water significantly increased blood uric acid levels in the mice (Supplementary Fig. 1). These results indicate that adenine is significantly toxic, whereas inosine, guanosine, and hypoxanthine induce mild hyperuricemia in mice.

### 3.2 Comparative analysis of renal and hepatic effects induced by different methods

**Fig. 2:**
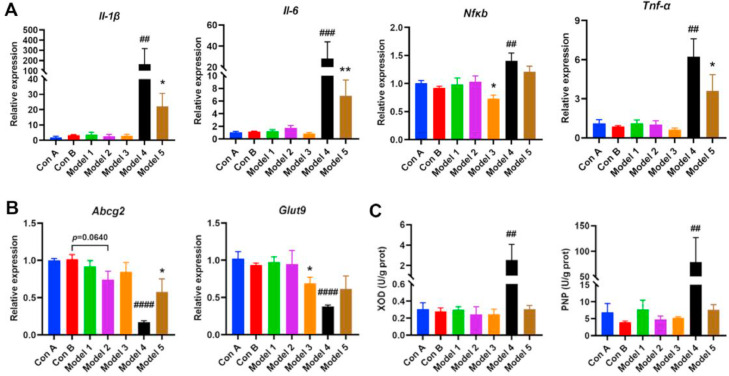
**Comparison of the effects of different HUA modeling methods on hepatic and renal functions.** (A) Relative mRNA expression of inflammatory cytokines in kidney. (B) Relative mRNA expression of ABCG2 and GLUT9 in kidney. (C) XOD and PNP activity in the liver. Data represent mean values ± SEM (*n* = 6 per group). Statistical analysis was performed using unpaired T test. The symbol “*” indicates comparison with Con A group. *p < 0.05, **p < 0.01. The symbol “**#**” indicates comparison with Con B group. **##***p* < 0.01, **###***p* < 0.001, **####***p* < 0.0001.

With respect to renal effects, significant differences were observed in the effects of various induction methods on kidney function. Compared with the Con B group, mice in the Model 4 group presented markedly increased serum creatinine and urea levels (Fig. 1D and E). Moreover, the serum creatinine level of mice in the Model 5 group was significantly greater than that of mice in the Con A group (Fig. 1D), suggesting that adenine may have induced renal dysfunction. Additionally, the renal indices of the mice in the Model 4 group were significantly greater than those of mice in the Con B group, and their renal tissues appeared yellow (Fig. 1F and G). Histological examination of renal tissues via H&E staining (Fig. 1G) revealed the most severe renal injury in the Model 4 group, characterized by detachment of renal tubular epithelial cells, exposure of tubular basement membranes, interstitial edema with inflammatory cell infiltration, proliferation of epithelial layers in the glomerular capsule wall to form crescents, and narrowing of the glomerular capsule lumen. The kidneys of the mice in the Model 2 and Model 5 groups also presented pathological damage, whereas those in the Model 1 and Model 3 groups presented no significant pathological changes (Fig. 1G). One mouse from each group was randomly selected to measure the glomerular filtration rate (GFR), and the results indicated that the GFR of the mice in the Model 4 group was only 0.18 mL/min/100 g body weight, suggesting severe impairment of renal filtration function (Fig. 1H). Furthermore, the GFR of the mice in the Model 2 and Model 5 groups also tended to decrease. The RT‒qPCR results revealed that cytokine transcript levels were significantly increased in the kidneys of the mice in the Model 4 and Model 5 groups, whereas the transcript levels of the uric acid transporter proteins ABCG2 and GLUT9 were decreased (Fig. 2A and B). These findings suggest that Model 4 and Model 5 resulted in severe renal impairment.

Additionally, we assessed the effects of different modeling methods on liver tissue. No significant differences in the serum levels of ALT and AST were detected between the mice in each model group and those in the control group (Supplementary Fig. 2A). The liver H&E staining results revealed no significant differences in the morphology or structure of the liver tissues in the model groups (Supplementary Fig. 2B). However, XOD and PNP activities were significantly increased in the liver tissues of mice in the Model 4 group. These results suggest that different modeling methods do not cause pathological damage to liver tissues, but that high doses of adenine promote uric acid synthase activity in the liver.

### 3.3 In vitro experiments analyzing the potential of probiotics for the treatment of hyperuricemia

**Fig. 3:**
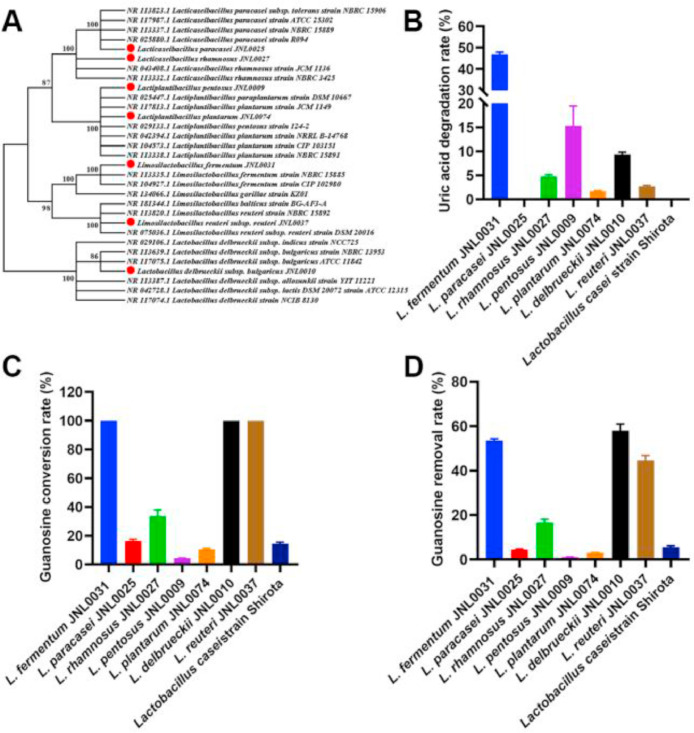
**In vitro experiments analyzing the potential of probiotics to modulate blood uric acid.** (A) Phylogenetic tree of probiotics constructed by comparing the 16S rRNA gene sequences based on Neighbor-Joining method. (B) Uric acid degradation rate. (C) Guanosine conversion rate. (D) Guanosine removal rate. Data represent mean values ± SEM (*n* = 3 per group).

Probiotics demonstrate potential advantages for treating hyperuricemia. In this study, seven strains of probiotics were isolated from fermented dairy products, and a phylogenetic tree was constructed by comparing the 16S rRNA gene sequences of the bacteria (Fig. 3A). The reabsorption of uric acid and purine metabolites in the intestine significantly impacts blood uric acid levels. Therefore, we evaluated the ability of these probiotics to degrade uric acid and guanosine using in vitro experiments. The results showed that L. *fermentum* JNL0031 was able to degrade up to 47 % of uric acid after incubation in a 1 mM uric acid solution for 2 h (Fig. 3B). L. *pentosus* JNL0009 and L. *delbrueckii* JNL0010 degraded 15.3 % and 9.4 % of the uric acid, respectively. L. *rhamnosus* JNL0027, L. *reuteri* JNL0037, and L. *plantarum* JNL0074 degraded 4.8 %, 2.7 % and 1.7 % of uric acid, respectively. L. *paracasei* JNL0025 and the L. *casei* strain Shirota did not degrade uric acid (Fig. 3B). Additionally, L. *fermentum* JNL0031, L. *delbrueckii* JNL0010, and L. *reuteri* JNL0037 achieved 100 % guanosine conversion rates and 53.6 %, 58.0 %, and 44.6 % guanosine removal rates, respectively, after incubation for 2 h in a 0.5 mM guanosine solution (Fig. 3C and D). These results suggest that these probiotics may reduce purine levels in the gut.

### 3.4 Effects of probiotics on a mouse model of hyperuricemia

**Fig. 4:**
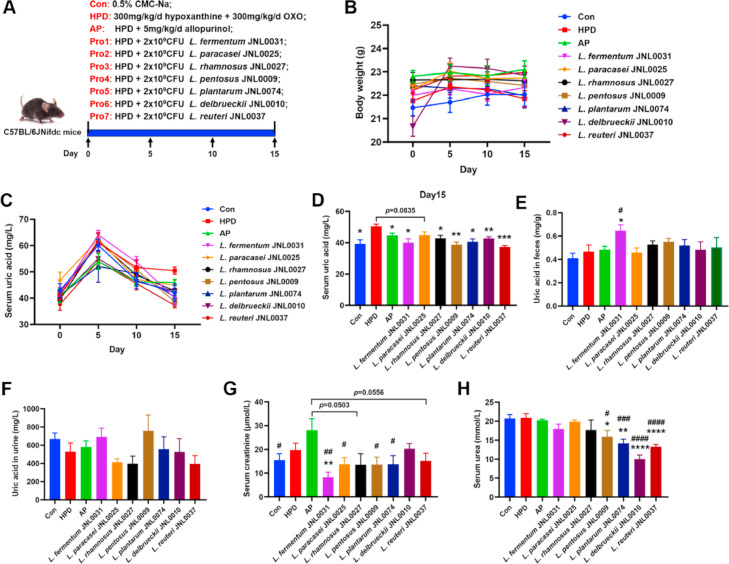
**Therapeutic effects of probiotics in HUA model mice.** (A) Schematic diagram of the study design. (B) Body weight. (C and D) Serum uric acid. (E) Levels of uric acid in feces. (F) Levels of uric acid in urine. (G) Serum creatinine. (H) Serum urea. Data represent mean values ± SEM (*n* = 7 per group). Statistical analysis was performed using unpaired T test. The symbol “*” indicates comparison with HPD group. *p < 0.05, **p < 0.01, ***p < 0.001, ****p < 0.0001. The symbol “**#**” indicates comparison with AP group. **#***p* < 0.05, **##***p* < 0.01, **###***p* < 0.001, **####***p* < 0.0001.

Next, we investigated the effects of these probiotics on the regulation of blood uric acid levels in a mouse model of hyperuricemia. In this study, the uric acid level in serum of mice from Model 3 was dramatically elevated, while no pathological changes were observed in the liver or kidneys, as demonstrated in 3.1, 3.2. Considering that intraperitoneal injection carries a higher likelihood of organ damage in comparison to gavage administration, Model 3 was selected for further experiments. We induced hyperuricemia in mice by gavage with hypoxanthine (300 mg/kg/d) and oteracil potassium (OXO, 300 mg/kg/d) for 15 consecutive days. Seven different strains of probiotics were administered to the mice by gavage on the same day as the start of modeling, and allopurinol treatment served as a positive control (Fig. 4A). Throughout the experiment, we observed that probiotic treatment did not significantly affect the body weights of the mice (Fig. 4B). On Day 15, the blood uric acid level in the HPD group was significantly greater than that in the Con group, whereas the blood uric acid level in the AP group was significantly lower than that in the HPD group (Fig. 4C and D). Probiotic treatments with L. *fermentum* JNL0031, L. *rhamnosus* JNL0027, L. *pentosus* JNL0009, L. *plantarum* JNL0074, L. *delbrueckii* JNL0010 and L. *reuteri* JNL0037 significantly reduced blood uric acid levels in mice compared with those in the HPD group (Fig. 4C and D). Additionally, L. *paracasei* JNL0025 tended to reduce blood uric acid levels (*p* = 0.0835). The effect of probiotics on uric acid levels in feces and urine of mice was further analyzed. The results showed that L. *fermentum* JNL0031 significantly increased the uric acid levels in feces compared with those of mice in the HPD and AP groups (Fig. 4E and F). There were no significant differences in fecal and urinary uric acid levels among the other treatment groups.

Furthermore, we analyzed the effects of probiotics on the creatinine and urea levels in the serum of the mice (Fig. 4G and H). The serum creatinine levels were significantly lower in the L. *fermentum* JNL0031 group than in the HPD group, whereas the urea levels were significantly lower in the L. *pentosus* JNL0009, L. *plantarum* JNL0074, L. *delbrueckii* JNL0010 and L. *reuteri* JNL0037 groups. The serum creatinine levels were significantly greater in the AP group than in the Con group. The serum creatinine levels were significantly lower in the L. *fermentum* JNL0031, L. *paracasei* JNL0025, L. *pentosus* JNL0009 and L. *plantarum* JNL0074 groups than in the AP group. Moreover, the serum levels of urea were significantly lower in the L. *pentosus* JNL0009, L. *plantarum* JNL0074, L. *delbrueckii* JNL0010 and L. *reuteri* JNL0037 groups than in the AP group (Fig. 4G and H). These results suggest that specific probiotics not only reduce blood uric acid levels in HUA mice, but also may have positive effects on renal filtration.

### 3.5 Effects of probiotics on uric acid metabolism and kidney tissue inflammation

**Fig. 5:**
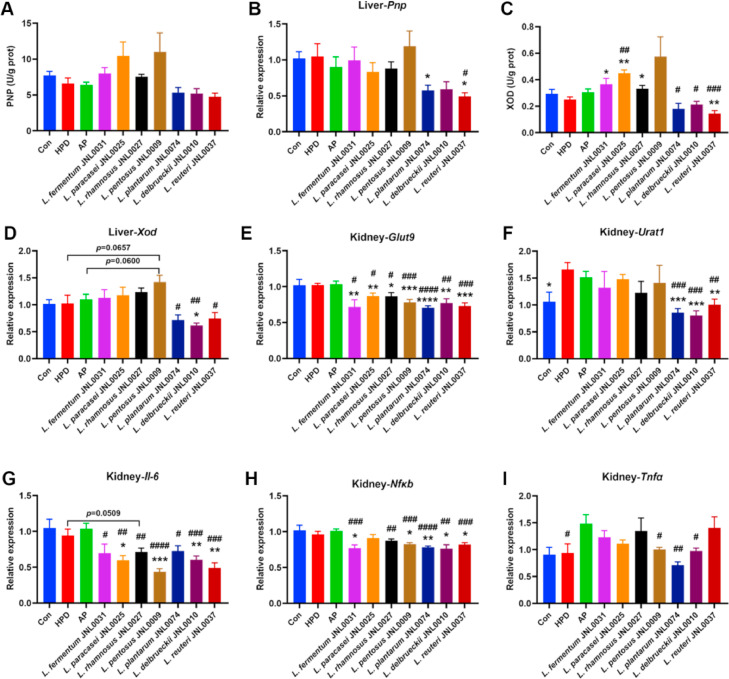
**Effects of probiotics on the function of liver and kidney in HUA model mice.** (A and C) PNP and XOD activity in the liver. (B and D) Relative mRNA expression of PNP and XOD in liver. (E and F) Relative mRNA expression of GLUT9 and URAT1 in kidney. (G, H and I) Relative mRNA expression of inflammatory cytokines IL-6, NF-κB and TGF-α in kidney. Data represent mean values ± SEM (*n* = 7 per group). Statistical analysis was performed using unpaired T test. The symbol “*” indicates comparison with HPD group. *p < 0.05, **p < 0.01, ***p < 0.001, ****p < 0.0001. The symbol “**#**” indicates comparison with AP group. **#***p* < 0.05, **##***p* < 0.01, **###***p* < 0.001, **####***p* < 0.0001.

Next, we analyzed the mechanism by which probiotics regulate blood uric acid levels. By examining uric acid synthase activity in the liver, we found no significant difference in PNP activity among the treatment groups (Fig. 5A). However, the transcript levels of hepatic PNP in the L. *plantarum* JNL0074 and L. *reuteri* JNL0037 treatment groups were significantly lower than those in the HPD group (Fig. 5B). In addition, hepatic XOD activity was significantly lower in the L. *reuteri* JNL0037 group than in the HPD group (Fig. 5C). The XOD activities and transcript levels in the L. *plantarum* JNL0074, L. *delbrueckii* JNL0010 and L. *reuteri* JNL0037 groups were significantly lower than those in the AP group (Fig. 5C and D). Interestingly, liver XOD activity was significantly greater in the L. *fermentum* JNL0031, L. *paracasei* JNL0025 and L. *rhamnosus* JNL0027 groups than in the HPD group (Fig. 5C). The small intestine and kidneys are the primary organs for uric acid excretion. RT‒qPCR analysis revealed no significant differences in the transcript levels of the uric acid transporter proteins GLUT9 and ABCG2 in the small intestine among the treatment groups (Supplementary Fig. 3A and B). However, the transcript levels of renal GLUT9 in the L. *fermentum* JNL0031, L. *paracasei* JNL0025, L. *rhamnosus* JNL0027, L. *pentosus* JNL0009, L. *plantarum* JNL0074, L. *delbrueckii* JNL0010 and L. *reuteri* JNL0037 groups were significantly lower than those in the HPD and AP groups (Fig. 5E). In addition, the transcript levels of renal solute carrier family 22 member 12 (*Slc22a12*, also known as URAT1) in the L. *plantarum* JNL0074, L. *delbrueckii* JNL0010 and L. *reuteri* JNL0037 groups were significantly lower than those in the HPD and AP groups (Fig. 5F). These results suggest that specific probiotics may regulate blood uric acid levels by modulating hepatic uric acid synthase activity as well as the renal transport of uric acid in mice.

Analysis of inflammation levels in mice revealed significantly lower renal transcript levels of IL-6 in the L. *paracasei* JNL0025, L. *pentosus* JNL0009, L. *delbrueckii* JNL0010, and L. *reuteri* JNL0037 groups than in the HPD group (Fig. 5G). The L. *fermentum* JNL0031, L. *pentosus* JNL0009, L. *plantarum* JNL0074, L. *delbrueckii* JNL0010, and L. *reuteri* JNL0037 groups presented significantly lower renal transcript levels of NF-κB than did the HPD group (Fig. 5H). Notably, the renal transcript levels of TNF-α were significantly greater in the AP group than in the HPD group, whereas the renal transcript levels of cytokines in the probiotic-treated group were significantly lower than those in the AP group (Fig. 5G-I). These results suggest that probiotics help reduce inflammation levels in the kidneys.

### 3.6 Effect of probiotics on the content of SCFAs in the gut

**Fig. 6:**
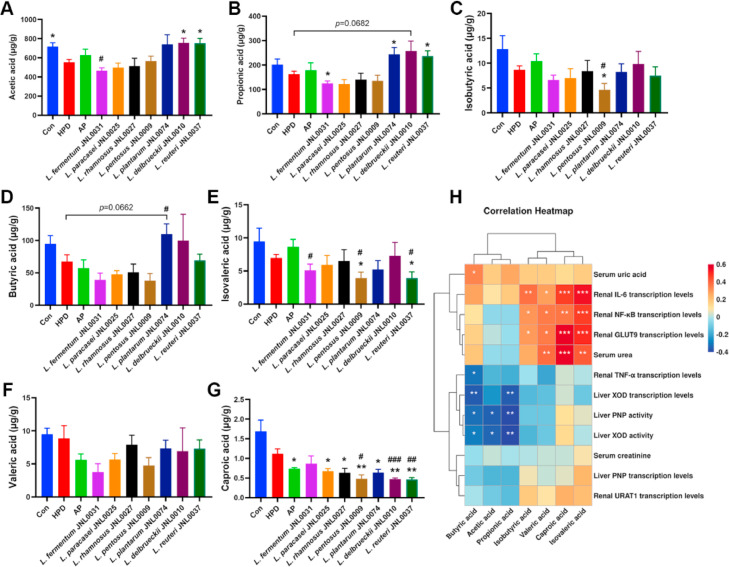
**Effect of probiotics on the level of SCFAs in colonic contents.** (A) Acetic acid. (B) Propionic acid. (C) Isobutyric acid. (D) Butyric acid. (E) isovaleric acid. (F) Valeric acid. (G) Caproic acid. (H) Correaltion heatmap. Data represent mean values ± SEM (*n* = 6 per group). Statistical analysis was performed using unpaired T test. The symbol “*” indicates comparison with HPD group. *p < 0.05, **p < 0.01. The symbol “**#**” indicates comparison with AP group. **#***p* < 0.05, **##***p* < 0.01, **###***p* < 0.001.

Probiotics can affect homeostasis by regulating the levels of SCFAs in the gut. In this study, the levels of acetic acid, propionic acid, butyric acid, isobutyric acid, valeric acid, isovaleric acid, and caproic acid in the colonic contents of mice were analyzed by GC–MS. The results revealed that, compared with the Con group, the HPD group exhibited a significant reduction in colonic acetic acid levels (Fig. 6A), with a downward trend observed in the levels of propionic, isobutyric, butyric, valeric, isovaleric and caproic acids (Fig. 6B-E and G). Compared with the HPD group, the propionic acid levels were significantly reduced in the L. *fermentum* JNL0031 group (Fig. 6B). The caproic acid levels were significantly lower in the L. *paracasei* JNL0025 and L. *rhamnosus* JNL0027 groups than those in the HPD group (Fig. 6G). The L. *pentosus* JNL0009 group had significantly lower levels of isobutyric, isovaleric, and caproic acids in the colonic contents compared to both the HPD and AP groups (Fig. 6C, E, and G). In the L. *plantarum* JNL0074 group, propionic acid levels were significantly higher than in the HPD group, along with a trend of increased acetic and butyric acid, and a significant decrease in caproic acid (Fig. 6A, B, D, and G). The L. *delbrueckii* JNL0010 group exhibited significantly higher acetic acid levels and significantly lower caproic acid levels compared with the HPD group (Fig. 6A and G), with a trend toward increased propionic acid levels (Fig. 6B). The L. *reuteri* JNL0037 group demonstrated significant increases in acetic and propionic acid levels and significant decreases in isovaleric and caproic acid levels compared with the HPD group (Fig. 6A, B, E, and G). Furthermore, Spearman correlation analysis indicated that the levels of isobutyric, valeric, isovaleric and caproic acids in the colonic contents were significantly and positively correlated with the serum urea and transcription levels of renal IL-6, NF-κB and GLUT9; while the levels of butyric, acetic and propionic acid were negatively correlated with liver XOD and PNP activity, and renal TNF-α (Fig. 6H). These results suggest that probiotics can modulate the levels of SCFAs in the gut, which may be associated with their renoprotective effects and inhibition of uric acid synthesis.

## 4 Discussion

In this study, we systematically compared various methods for inducing hyperuricemia in C57BL/6JNifdc mice and explored the potential of probiotics to regulate blood uric acid levels. Our findings revealed that adenine causes acute renal tissue injury, whereas inosine, guanosine, and hypoxanthine can induce mild hyperuricemia without causing hepatic or renal tissue damage. Furthermore, our experiments confirmed that probiotics have the potential to regulate blood uric acid levels and exert a protective effect by inhibiting the expression of inflammatory cytokines and uric acid transporter proteins in the kidneys. These findings increase our understanding of HUA pathogenesis and provide valuable insights into the therapeutic potential of probiotics.

Drug-induced animal models of HUA are the most commonly used tools for studying the pathogenesis of HUA and therapeutic drugs. However, a wide range of animal strains have been utilized, including Kunming mice (D. Li et al., 2023), C57BL/6 mice (Wan et al., 2016), BALB/c mice (Wang et al., 2019), ICR mice (An et al., 2023), KK-Ay mice (Lu et al., 2020), SD rats (Zhu et al., 2022) and Arbor Acres broilers (D. Li et al., 2023). Different animal strains may respond differently to drugs, increasing the complexity of assessing the effects of therapeutic agents. C57BL/6 mice are particularly favored by researchers because of their uniform genetic background and immunological properties. Since the genetic background of genetically induced HUA mouse models is primarily C57BL/6 J, investigating methods for constructing stable HUA models on the basis of C57BL/6 J mice is essential.

Uric acid is a metabolite of purines such as adenine and guanine. In the liver, adenine is converted to hypoxanthine by adenine deaminase. Hypoxanthine is then converted to xanthine by xanthine oxidoreductases, including xanthine oxidase and xanthine dehydrogenase. Guanine is converted to xanthine by guanine deaminase. Xanthine is subsequently converted to uric acid by xanthine oxidoreductase. In mice, uric acid is converted to freely soluble allantoin by uricase and is then excreted through the kidneys. However, the gene encoding uricase is nonfunctional in humans (Kratzer et al., 2014). Drug-induced mouse models of HUA have been developed primarily by increasing uric acid synthesis while inhibiting uricase activity via the use of oteracil potassium. The precursor drugs of uric acid used in the literature to induce HUA include inosine, guanosine, adenine, hypoxanthine, and yeast extracts. There are also studies that have used intraperitoneal injections or dietary additions of uric acid. Our study revealed that adenine induced severe renal injury, which is consistent with previous findings (Wen et al., 2020). However, asymptomatic hyperuricemia typically does not cause renal injury (Sellmayr et al., 2020). Moreover, most patients with clinical hyperuricemia do not develop renal injury (Obermayr et al., 2008; Weiner et al., 2008). Therefore, we believe that adenine may not be suitable for inducing a simple model of hyperuricemia. In addition, we attempted to establish an HUA mouse model via the gavage of uric acid and oteracil potassium, but the blood uric acid levels of the model mice did not significantly increase compared with those in the control group. This may be due to the inability of the intestines to fully absorb exogenous uric acid and the fact that anaerobic bacteria in the intestines can directly break down uric acid (Liu et al., 2023), preventing the maintenance of high blood uric acid concentrations. Some studies have reported a dose-dependent relationship between high-fructose corn syrup intake and serum uric acid levels, and intravenous glucose infusion can significantly increase serum uric acid concentrations (Helget and Mikuls, 2022; Raivio et al., 1975). Mechanistically, a high-fructose diet increases uric acid production by accelerating the degradation of adenosine triphosphate to adenosine monophosphate, the precursor of uric acid. High-fructose diets also induce metabolic disorders such as obesity and fatty liver disease (Hannou et al., 2018), so the influence of sugar and lipid metabolism on uric acid metabolism needs to be considered. Interestingly, the blood uric acid levels tended to increase but then decreased with all the methods we compared. The inability of mice to maintain high blood uric acid levels may be due to the protective effects exhibited by the organism. This finding also serves as a reminder to researchers to consider the optimal window for evaluating the effectiveness of drug therapies for HUA.

Probiotics have various mechanisms for regulating uric acid. Our results revealed that several strains, including L. *fermentum* JNL0031, L. *delbrueckii* JNL0010 and L. *reuteri* JNL0037, efficiently degrade uric acid and guanosine. These findings are consistent with those of previous studies. Li *et al*. reported that the absorption of nucleobases by intestinal epithelial cells was less than that of nucleosides. *Lactiplantibacillus plantarum* is able to reduce the uptake of substrates for uric acid synthesis by degrading nucleosides to purines via ribonucleoside hydrolases (D. Li et al., 2023). Moreover, the gut microbiota encodes genes that degrade uric acid and can convert uric acid into xanthine or SCFAs (Kasahara et al., 2023; Liu et al., 2023; R. Zhao et al., 2022). Additionally, our results suggest that certain probiotic strains may regulate blood uric acid levels by modulating hepatic uric acid synthase activity and renal uric acid transport. L. *reuteri* JNL0037 significantly inhibited hepatic XOD activity, suggesting that probiotics can inhibit uric acid synthesis. Probiotics also significantly inhibited the transcript levels of the *Glut9* and *Urat1* genes in the kidneys. GLUT9 and URAT1 are major proteins involved in the reabsorption of uric acid in the kidneys (Major et al., 2024; Vitart et al., 2008), suggesting that probiotics may inhibit the reabsorption of uric acid in the kidneys, thereby lowering serum uric acid levels.

In addition to directly regulating uric acid synthesis and metabolic processes, probiotics may also play uric acid-lowering and renoprotective roles by influencing the structure of the gut microbiota and metabolite levels. Studies have shown that many intestinal commensal bacteria encode enzymes that degrade purine-like substances and are able to convert uric acid into lactic acid or short-chain fatty acids (Kasahara et al., 2023; Liu et al., 2023). Some metabolites of gut commensal bacteria are capable of modulating the expression of intestinal uric acid transporter proteins. For instance, hippuric acid, a metabolite produced by *Alistipes indistinctus*, enhances the expression of intestinal ABCG2, thereby promoting the excretion of uric acids (Xu et al., 2024). In addition, probiotics are able to maintain gut microbiota homeostasis, increase the abundance of beneficial bacteria (e.g., *Lactobacillus* and *Bifidobacterium*, etc.), and reduce the proportion of pro-inflammatory bacteria, as well as attenuate oxidative stress and inflammatory responses in the body, and repair renal tissue damage (Huang and Chen, 2024; Zhu et al., 2021). In this study, the reduction in inflammatory cytokines in the kidneys of probiotic-treated mice indicates the renoprotective effects of these strains. In future studies, we will analyze the mechanism of gut microbiota in the role of probiotics in improving renal tissue function.

While the current study provides valuable insights into the hyperuricemia mouse model and the potential therapeutic effects of probiotics, there are several limitations that warrant acknowledgment. First, our study utilized a murine model to investigate the effects of different induction methods and probiotics on hyperuricemia. Although mice are commonly used for such investigations, they may not fully replicate the complexity of human physiology and metabolism, particularly in terms of uric acid handling and the impact of the gut microbiota. These species-specific differences could affect the translation of our findings to clinical settings. Second, the study primarily assessed the short-term effects of probiotics on blood uric acid levels and renal function. Long-term effects, including the sustainability of probiotic effects and potential adaptation of the gut microbiota over time, have not been addressed. This limitation is significant because it pertains to the chronic nature of hyperuricemia and the need for long-term management strategies. Additionally, while we observed changes in the expression of uric acid transporter proteins and inflammatory markers, the precise molecular mechanisms by which probiotics exert their effects remain to be fully elucidated. Future studies should aim to dissect these mechanisms at the cellular and molecular levels, potentially using techniques such as transcriptomics, proteomics, and metabolomics. We also have noted that the initial gut microbiota composition of mice before probiotic treatment should be fully considered, as it may influence the response of model mice to probiotic interventions.

## 5 Conclusions

Our comprehensive analysis of different hyperuricemia induction methods in mice highlights the need for standardized models that accurately reflect the disease pathophysiology. The varying degrees of renal impairment and the transient nature of hyperuricemia observed with some methods underscore the importance of model selection in preclinical research. Furthermore, our investigation of probiotic interventions reveals their potential as effective regulators of blood uric acid levels. Specific probiotic strains, such as L. *fermentum* JNL0031 and L. *reuteri* JNL0037, showed promising effects in reducing uric acid levels and mitigating renal inflammation. Based on our research and published studies (Cheng et al., 2025), probiotics exert uric acid-lowering and renoprotective effects through several mechanisms: (1) probiotics directly degrade uric acid and its precursors, thereby reducing gut absorption and accumulation; (2) probiotics reduce uric acid synthesis by inhibiting key enzyme activity; (3) probiotics modulate the expression of uric acid transporters in the kidneys and intestines, promoting uric acid excretion and inhibiting its reabsorption; (4) probiotics increase beneficial bacteria and decrease pro-inflammatory bacteria, thereby enhancing the balance of gut microbiota; (5) probiotics produce metabolites that mitigate oxidative stress and inflammatory responses in the body. These results suggest that probiotics could serve as a safe and viable alternative or adjunct to traditional pharmacological treatments for hyperuricemia. Future studies should focus on elucidating the precise mechanisms of action and evaluating the long-term efficacy and safety of probiotics in clinical settings.

**All authors commit to the authenticity of the research data.**

## CRediT authorship contribution statement

**Yanbo Wang:** Conceptualization, Data curation, Funding acquisition, Writing – original draft. **Huijiao Zhang:** Data curation, Investigation, Project administration. **Shujun Liu:** Data curation, Investigation, Project administration. **Sheng Sun:** Methodology, Resources. **Weibin Ren:** Methodology, Validation. **Tao Wang:** Methodology, Validation. **Shujuan Zhang:** Resources, Data curation. **Hangping Yao:** Supervision, Writing – review & editing. **Changzhong Jin:** Supervision, Writing – review & editing. **Nanping Wu:** Conceptualization, Funding acquisition, Writing – review & editing.

## Declaration of competing interest

The authors declare that they have no known competing financial interests or personal relationships that could have appeared to influence the work reported in this paper.

## Data Availability

Data will be made available on request.

## Appendix Supplementary materials

Image, application 1
