# Supplementary material for: Comparative analysis of hyperuricemia induction methods and probiotic interventions in mice
Source: Curr Res Microb Sci. 2025 Jun 12;9:100422. doi: 10.1016/j.crmicr.2025.100422 (PMC12221599; doi:10.1016/j.crmicr.2025.100422)
Supplement: Supplementary file 1 [file mmc1.docx]

**Title: Comparative Analysis of Hyperuricemia Induction Methods and Probiotic Interventions in Mice**

**Authors: Yanbo Wang^1^, Huijiao Zhang^1^, Shujun Liu^1^, Sheng Sun^2^, Weibin Ren^1^, Tao Wang^1^, Shujuan Zhang^1^, Hangping Yao^1,3^*, Changzhong Jin^1,3^* and Nanping Wu^1^***

**Affiliations:** 1. Jinan Microecological Biomedicine Shandong Laboratory, Jinan 250117, China; 2. Changchun Veterinary Research Institute, Chinese Academy of Agricultural Sciences, Changchun 130122, China; 3. State Key Laboratory for Diagnosis and Treatment of Infectious Diseases, The First Affiliated Hospital, College of Medicine, Zhejiang University, No. 79 Qingchun Road, Hangzhou 310003, Zhejiang Province, China.

Y. Wang, H. Zhang and S. Liu contributed equally to this work.

*Corresponding authors: [flwnp2013@163.com](mailto:flwnp2013@163.com) (N. Wu), [changzhongjin@163.com](mailto:changzhongjin@163.com) (C. Jin) and [yaohangping@zju.edu.cn](mailto:yaohangping@zju.edu.cn) (H. Yao).

**Table S1** Primers used in this paper

| Gene | Forward primer (5’-3’) | Reverse primer (5’-3’) | Reference |
| --- | --- | --- | --- |
| *β-actin* | GGCTGTATTCCCCTCCATCG | CCAGTTGGTAACAATGCCATGT | This study |
| *Il-1β* | CCAACAAGTGATATTCTCCATGAG | ACTCTGCAGACTCAAACTCCA | (Jin *et al.*, 2018) |
| *Il-6* | CTGCAAGAGACTTCCATCCAG | AGTGGTATAGACAGGTCTGTTGG | This study |
| *Nf-κb* | CCTGCAGGGTCACTCGATTT | TCAGAACCAAGAAGGACGCC | (Jin *et al.*, 2018) |
| *Tnf-α* | GTCTACTGAACTTCGGGGTGAT | GGCTACAGGCTTGTCACTCG | (Jin *et al.*, 2018) |
| *Abcg2* | GAACTCCAGAGCCGTTAGGAC | CAGAATAGCATTAAGGCCAGGTT | This study |
| *Glut9* | TTGCTTTAGCTTCCCTGATGTG | GAGAGGTTGTACCCGTAGAGG | This study |
| *Xod* | TGATGGTTCGGTGCTGTTGA | GGGACGGTGTTAGTGCTTGT | (Li *et al.*, 2023) |
| *Pnp* | GCCTGGAAACAAATGGGG | ACCAAAGACACGGAGCCC | (Li *et al.*, 2023) |
| *Urat1* | CCGCTTCCGACAACCTCA | CTTCTGCGCCCAAACCTATC | (Li *et al.*, 2023) |
| *Npt1* | TGACACTTGCCAACTCAACACT | CCAAACATTCCAATTAAAGCTG | (Li *et al.*, 2023) |


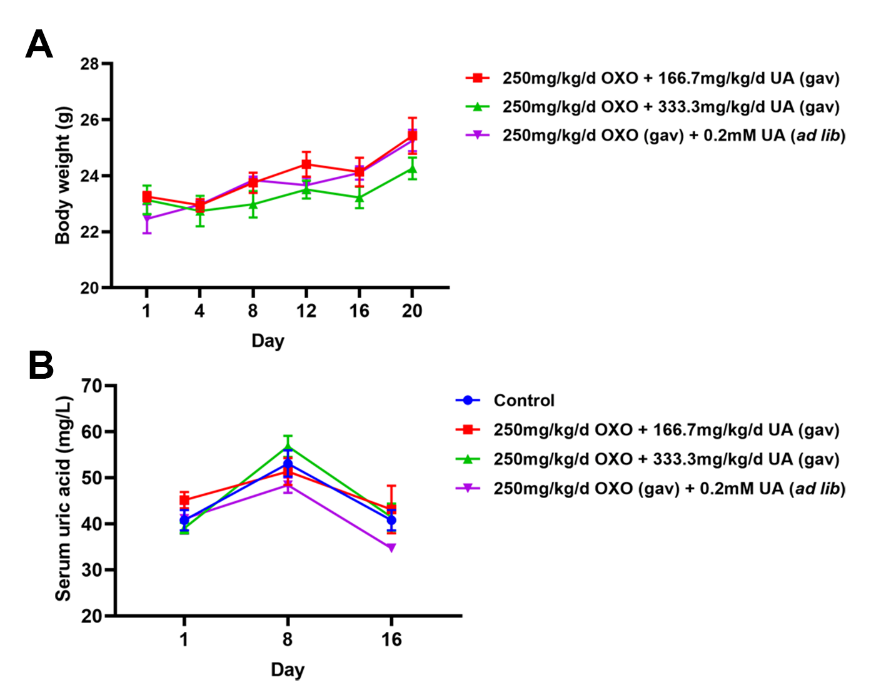


**Supplementary Figure 1 Comparison of blood uric acid levels in different HUA modeling methods.** (A) Body weight. (B) Serum uric acid. Data represent mean values ± SEM (n = 5 per group). Statistical analysis was performed using unpaired T test.


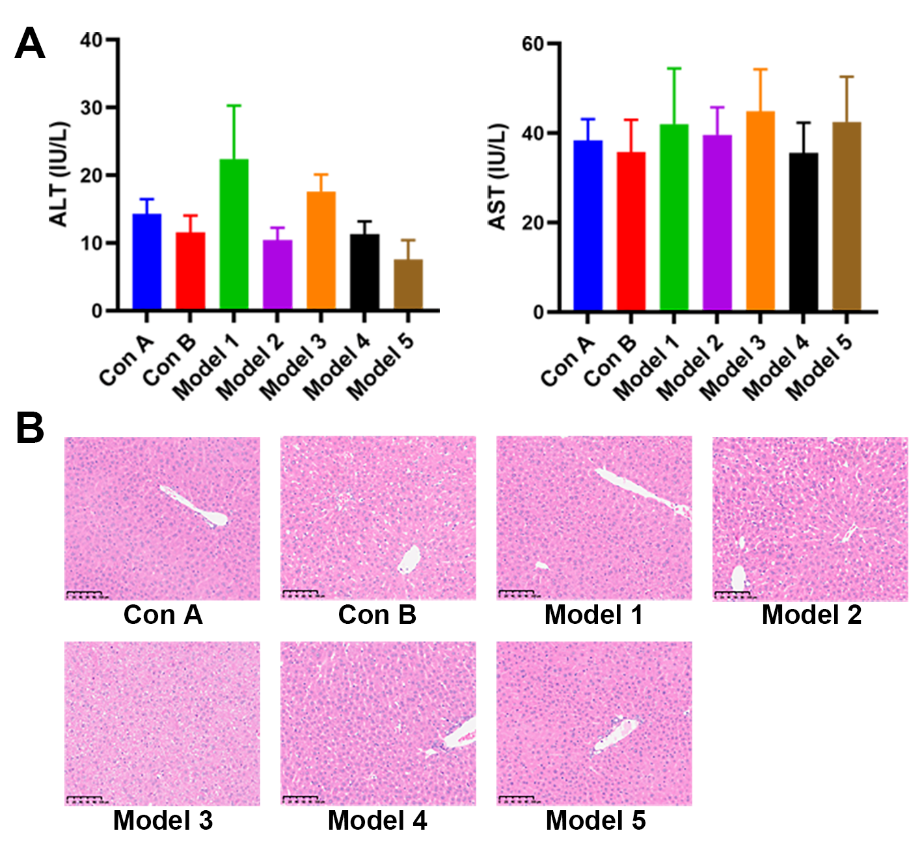


**Supplementary Figure 2 Comparison of the effects of different HUA modeling methods on hepatic functions.** (A) Serum ALT and AST. (B) H&E staining of the liver. Data represent mean values ± SEM (n = 5 per group).


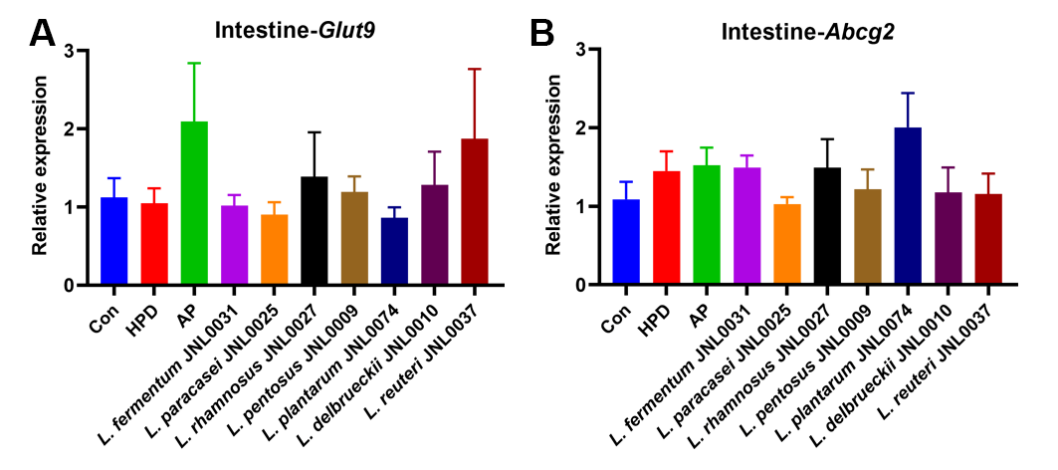


**Supplementary Figure 3 Effects of probiotics on the function of small intestine in HUA model mice.** (A and B) Relative mRNA expression of GLUT9 and ABCG2 in small intestine. Data represent mean values ± SEM (n = 7 per group). Statistical analysis was performed using unpaired T test.

**References**

Jin, C., Z. Zeng, C. Wang, T. Luo, S. Wang, J. Zhou, Y. Ni, Z. Fu and Y. Jin, 2018. Insights into a possible mechanism underlying the connection of carbendazim-induced lipid metabolism disorder and gut microbiota dysbiosis in mice. Toxicol. Sci., 166(2): 382-393. Available from <https://www.ncbi.nlm.nih.gov/pubmed/30496565>. DOI 10.1093/toxsci/kfy205.

Li, M., X. Wu, Z. Guo, R. Gao, Z. Ni, H. Cui, M. Zong, F. Van Bockstaele and W. Lou, 2023. Lactiplantibacillus plantarum enables blood urate control in mice through degradation of nucleosides in gastrointestinal tract. Microbiome, 11(1): 153. Available from <https://www.ncbi.nlm.nih.gov/pubmed/37468996>. DOI 10.1186/s40168-023-01605-y.
